# Book Reviews

**Published:** 1874-04-15

**Authors:** 


### Book Reviews.

TREATMENT of Nervous and Rheumatic Affections by Static Electricity. By Dr. Arthius. Translated from the French by J. H. Etheridge, M.D., Professor of General Therapeutics, Rush Medical College, Chicago.

It is difficult to review with perfect fairness, and to criticise with entire impartiality, the work of a friend. It is more difficult to expose completely the errors and absurdities of a work, without involving the appearance of personal hostility to the author. The truth of these remarks impresses us now with great force as the above-mentioned book lies before us. Actuated by feelings of the warmest friendship for the translator, we have read the book over and over again, in the hope of finding something which we could honestly commend in it. But we have been grievously disappointed. There is nothing admirable of the work but its exceeding small size. When we consider that the translation was made by Dr. Etheridge "from a desire to contribute to the literature of a subject scarcely known to young American physicians," we are filled with regret that the limited means at his command were so inadequate to his purpose. How sad it is that such a desire, when it became perfectly irresistible, was not tempered with a proper regard for his own reputation, and for the feelings of the American profession!

Though we firmly believe his intentions are good, we deplore his inability to distinguish between a genuine contribution to knowledge and a shallow advertisement of an ignorant quack. Our language is strong; but that it is actually milder than the facts warrant, a perusal of the book will show. From the "Introduction" we extract the following passages: "Of all the treatments of nervous diseases and rheumatic affections now in vogue, none is to be compared, for efficacy, to the electrical treatment, which *we advocate*." * * * "Dynamic electricity (the form usually employed) is often dangerous, rarely efficacious." * * * "Static electricity, on the contrary, *our* electricity, cannot in a single case be dangerous; even when it is not curative, it is beneficial. This is pre-eminently a regulator of the functions, a dispenser of harmony, a distributor of equilibrium."

We would suggest to the author that if he can find time to attend the next meeting of the Illinois State Medical Society, he may hear of something to his advantage from Dr. D. Prince. The book abounds in such language as we have quoted; indeed, it is the only compensation afforded for dearth of ideas.

The first chapter is devoted to a superficial, fragmentary, and imperfect historical review of the subject. In the second chapter, an absurd attempt is made to expose the "Inferiority of Dynamic Electricity: its Dangers." Though the language in this is highly sensational and exaggerated, the author unquestionably shows that "dynamic electricity" may, when improperly used, do considerable damage.

Some idea of the clearness of his knowledge may be acquired from the following quotation: "But it must be immediately said, these currents (of dynamic electricity) derive their origin and power from chemical decomposition." * * * "Dynamic electricity participates, necessarily, in the very elements which compose the generating pile." We suppose this is all true, though we have not a very clear idea of the author's meaning. We are afraid, however, that his perception is somewhat obscured by an erroneous idea of the capacity of the agent, and a morbid apprehension of its destructive tendency, when he says: "It is easy to understand all the disorders which can be produced (by dynamic electricity) in an organism as delicate as ours, all the corrosive currents, saturated with violent acids, which destroy everything from flesh even to metals." The book is full of just such twaddle. And this is the ground of our belief that the author is an ignoramus or a knave—probably both. The third chapter is descriptive of apparatus and mode of proceeding. The machine employed is distinguished from those in ordinary use by several striking peculiarities. The plate is 28½ inches in diameter. The rubbers are coated either with oxide of gold or ducto-sulphide of tin—preference being given to the former. The use of dissimilar metals in the construction of the apparatus is decried, on the ground that when they are used a portion of each finds entrance into the body with the electric current, and by inharmonious action either fails in producing the best attainable result, or actually produces most singularly complicated and dangerous commotions. The conductor and excitator should always be of the same material; and the material in the excitator should vary with the disease. In commencing treatment, an attempt is made to determine by experiment the material that best accords with the requirements of the case. The insulator is a stool with glass feet, upon which the patient is required to sit. The "flindique bath" consists in charging the patient seated on the stool. Its efficacy is highly extolled. Treatment is invariably commenced with its use; and though it may accomplish no good, it cannot by any possibility do harm. Our author speaks of the pores of the skin "sucking up" electricity, and delights us with a charming disquisition on "electrical frictions," "douches," and "shampooings"—processes which are as much entitled to the names they receive as an enema would be. We are further informed "that experience has demonstrated that the electric current ought always to be directed from the head or spinal cord to the extremities;" and we accordingly find that he always connects the conductor with the head or spinal column of his patient. The author seems to ignore the history and physical signs of disease as a means of acquiring a knowledge of its nature and seat. The machine obviates all trouble and uncertainty; it makes the diagnosis for him. There is no need of patient inquiry and careful reasoning, and there is no cause for anxiety. "Whenever a man is suffering, the electric fluid ceases to pass uniformly in his organism." * * * "The patient perceives the slightest prickling in the sound parts, and none at all in the diseased parts." The seat of disease is thus readily determined. Never mind its nature: just use

"Static Electricity—*our* Electricity." The fourth and fifth chapters are devoted to the consideration of the "Transport of Medicines by Static Electricity."

It is claimed that a portion of the substances traversed by the electricity is conveyed, with the latter, into the body; and that the effects of all medicines may be procured by this mode of administration. This is suggestive; it is good. It is a greater contribution to medicine than is "Esmarck's Method" to surgery. Who can conceive of all the benefits that may be derived from this mode of practice? Here we have a parturient woman bleeding to death, in consequence of inertia of the uterus. We get out the machine; apply the conductor, well coated with fluid extract of ergot, to the spinal column, and touch the hypogastrium with the excitator; when lo! the uterus contracts, the hæmorrhage is stopped, and the baby is born! A patient has a most obstinate constipation. We *know* he has constipation—not because he said so, nor because we have watched him, but because he experienced no prickling in the abdomen when placed on the insulator, and connected with the machine. We put that patient on an insulated bed or sofa, taking great care that the lower part of his body points toward a slop-bucket—or, at least, away from us. We then apply the conductor, well smeared with castor oil, to the patient's pharynx, and when all arrangements are completed, touch the excitator, covered with a good *drawing salve*, to his anus — and his difficulty is removed with neatness and despatch. But, seriously, we do not doubt the possibility of introducing infinitessimal quantities of medicinal substances into the body in this way. To make it the basis of a new departure in therapeutics, however, would be almost as absurd as some of our author's diagnoses. To these we now invite attention [The following history is condensed, but differs from the original in no other respect]:

A child, two years of age, was attacked with some disease of the brain, immediately after having fallen to the ground from the arms of its nurse. There was incomplete recovery from the disorder, a tendency to convulsions remaining; and the severity of these convulsions, and the frequency of their occurrence, so increased, that, in time, the patient could secure only a very few hours of continuous repose. The ordinary remedies had been prescribed without success, and treatment was for a time abandoned. As the child advanced in age, the convulsions became somewhat less frequent; but its right arm became completely paralyzed, and much atrophied. Eight years after paralysis was established, and ten years after the injury was received, the patient was introduced to our author. His diagnosis was *epilepsy;* prognosis, favorable; treatment, static electricity; result, perfect recovery.

In his remarks on locomotor-ataxia, we find the following cheerful assurances: "The fatal designation, *progressive*, which M. Duchenne gave to it, does not belong to it to-day, since we are always able to arrest it in its invading stage. We have treated a large number of ataxias, and we are always successful in imposing a barrier to the disease."

In one case, the astounding diagnosis of "Rheumatism, General Debility and Moral Prostration," was made. But our author rose equal to the emergency, and in a jiffy relieved his patient of pain, and restored his original vigor and pristine purity. Though we have already exceeded the ordinary limits of a book review, we have exposed only a few of the absurdities of this work.

A reference to the original work shows that the author's ideas have been very fairly and faithfully translated. We notice, however, that his name, in French, is spelt, Arthuis. Arthius is, perhaps, Dr. Etheridge's English version.

During the reign of the late Emperor, the laws regulating the practice of medicine in France were very stringently enforced. No person was allowed to assume the title of, nor to practice as, a physician, until he had spent the required seven years of study, and received his degree at some French school of medicine. Under the present regimen, however, these very just and wholesome restrictions have been, to a great extent, withdrawn. From all quarters, the profession of France are sending up their protests against the swarm of unprincipled charlatans by whom their country is being overrun.

The simple title of Dr., which an author may prefix to his name, on a title page, is no longer, therefore, in France, more than it would be in our own country, a guarantee of professional standing.

In the absence, then, of any evidence whereby to judge of Dr. Arthuis' professional character and attainments, otherwise than from the general style and character of the work before us, we are, as before intimated, very much afraid that the translator has placed himself in the unfortunate position of endorsing, and placing before the American profession, the puff, quack advertisement, of some uneducated, unrecognized, foreign charlatan.

---

The Obstetrical Journal of Great Britain and Ireland, including Midwifery and Diseases of Children, with an American Supplement; Edited by Wm. F. Jenks, M.D. Philadelphia: Henry C. Lea.

The number for March closes the first volume of this excellent journal. Each number throughout the volume has contained a well-selected and choice variety of practical essays, lectures, translations, etc., appertaining to the special departments to which it is devoted. The American Supplement is well conducted, and adds much to the value of the issues.

The subscription price is $5.00 per annum, in advance. Any of our subscribers who desire can obtain it through our office at $4.00.

---

ANOTHER NEW JOURNAL.—We have received the first number of the *Missouri Clinical Record*, published at St. Louis, monthly, at $3.00 per annum. The number before us presents a fairly creditable appearance—contains sixteen quarto pages of reading matter, and includes in the contents a number of valuable original lectures. Communications, etc., should be addressed to the editor, W. A. Hardway, M.D., at Missouri Medical College, St. Louis, Missouri.

